# In-Hospital Outcomes of Thrombolysed Versus Late-Presenting Non-Thrombolysed Patients With Acute ST-Segment Elevation Myocardial Infarction

**DOI:** 10.7759/cureus.96623

**Published:** 2025-11-11

**Authors:** Mudasir Habib, Muzdalfa Parvez, Nouman Khan, Muhammad Afnan, Fakhar Zaman, Amjad Ali, Shakir Ullah

**Affiliations:** 1 Department of Cardiology, District Headquarters (DHQ) Teaching Hospital, Kohat Development Authority (KDA), Kohat, PAK; 2 Department of Cardiology, Khyber Teaching Hospital, Peshawar, PAK; 3 Department of Cardiology, Hayatabad Medical Complex, Peshawar, PAK; 4 Department of Internal Medicine, District Headquarters (DHQ) Teaching Hospital, Kohat Development Authority (KDA), Kohat, PAK

**Keywords:** delayed presentation, in-hospital mortality, myocardial infarction, reperfusion, st-elevation, thrombolytic therapy

## Abstract

Background: Early thrombolytic therapy substantially improves outcomes in patients with acute coronary syndrome (ACS), yet its timely administration remains suboptimal in many developing regions.

Objective: To evaluate in-hospital outcomes of patients with acute ST-elevation myocardial infarction (STEMI) receiving timely thrombolysis compared with those managed with late or no thrombolysis.

Methods: This comparative observational study included 136 consecutive ACS patients, divided equally into thrombolysis (n = 68) and late/non-thrombolysis groups (n = 68). Demographic data, risk factors, clinical presentation, and in-hospital outcomes, including mortality, high WHO CVD risk at presentation, recurrent ischemia, bleeding, and resource utilization, were collected. Data were analyzed using SPSS version 26 (IBM Corp., Armonk, NY), including t-tests, chi-square or Fisher’s exact tests, and multivariate logistic regression to adjust for confounding variables. Significance was set at p < 0.05.

Results: Patients receiving thrombolysis showed significantly lower mortality (2 (2.9%) vs 12 (17.6%), p = 0.01), heart failure (10 (14.7%) vs 28 (41.2%), p < 0.001), cardiogenic shock (4 (5.9%) vs 14 (20.6%), p = 0.01), and recurrent ischemia (4 (5.9%) vs 14 (20.6%), p = 0.01). High WHO cardiovascular risk at presentation was also less frequent among thrombolyzed patients (12 (17.6%) vs 30 (44.1%), p < 0.001). Bleeding complications were rare and comparable (6 (8.8%) vs 8 (11.8%), p = 0.57), supporting the safety of timely thrombolysis. Multivariate logistic regression confirmed that thrombolysis independently reduced composite adverse outcomes (adjusted OR = 0.42; 95% CI 0.25-0.70; p = 0.001).

Conclusion: Timely thrombolytic therapy significantly improves in-hospital outcomes and can be safely administered in ACS patients when delivered within recommended time windows.

## Introduction

Acute ST-segment elevation myocardial infarction (STEMI) represents one of the most critical manifestations of coronary artery disease, resulting from the complete occlusion of a coronary artery that, without timely reperfusion, leads to extensive myocardial ischemia and necrosis [1]. Globally, cardiovascular diseases account for nearly one-third of all deaths, and STEMI remains a major contributor to this burden [2]. All acute coronary syndrome cases present as STEMI, with a rising incidence among younger adults due to the increasing prevalence of hypertension, diabetes, and smoking. The condition imposes substantial health and economic costs, particularly in low- and middle-income countries (LMICs), where access to advanced cardiac care remains limited [3].

Prompt reperfusion is the central goal of STEMI management. It refers to the restoration of coronary blood flow within evidence-based timelines - within 90 minutes for primary percutaneous coronary intervention (PCI) and within 12 hours for fibrinolytic (thrombolytic) therapy [4,5]. Achieving reperfusion within these “golden hours” minimizes myocardial injury, preserves left ventricular function, and reduces mortality [6]. In resource-limited settings where timely PCI is not universally available, intravenous thrombolysis - most commonly with streptokinase - remains the most practical and life-saving alternative [7].

However, delayed hospital presentation remains a major barrier to effective STEMI care in developing countries. Factors contributing to such delays include lack of public awareness about cardiac symptoms, financial constraints, limited access to emergency transport, and systemic inefficiencies in referral pathways [8,9]. Pathophysiologically, delayed reperfusion allows irreversible myocardial necrosis to progress, increasing infarct size, worsening left ventricular remodeling, and predisposing patients to arrhythmias, cardiogenic shock, and post-MI heart failure [10].

While the efficacy of early thrombolysis is well established in clinical trials and large registries, there remains a paucity of real-world comparative data evaluating in-hospital outcomes between patients who receive timely thrombolysis and those who present too late to benefit, especially in LMIC contexts [11]. Such evidence is critical for guiding national cardiac care strategies, refining emergency response systems, and improving patient education to reduce pre-hospital delays [12].

This study was therefore designed to compare in-hospital outcomes between STEMI patients who received timely thrombolytic therapy and those who presented late and did not receive thrombolysis. We hypothesized that prompt thrombolysis would be associated with significantly lower in-hospital mortality, fewer complications (such as heart failure and recurrent ischemia), and shorter hospital stays compared to late-presenting patients. By focusing on in-hospital outcomes, this study provides an essential snapshot of the immediate impact of reperfusion timing on morbidity and resource utilization, data that are highly relevant for improving acute cardiac care pathways in resource-constrained healthcare systems.

## Materials and methods

Study design and setting

This comparative cross-sectional study was conducted at the Department of Cardiology, District Headquarters (DHQ) Hospital KDA Kohat, Khyber Pakhtunkhwa, Pakistan, over a 12-month period from 1st January 2024 to 31st December 2024. The study adhered to standard clinical and ethical research protocols for observational analyses.

Sample size calculation

Based on post-infarct angina rates reported in a previous study [13] (2.7% in thrombolysed patients vs. 22.9% in late-presenting non-thrombolysed patients), the required sample size was calculated using the two-proportion formula for a two-sided test with α=0.05\alpha = 0.05α=0.05 and 95% power [14]: $n = \frac{\left[ Z_{1-\alpha/2} \sqrt{2 \bar{p} (1-\bar{p})} + Z_{1-\beta} \sqrt{p_1 (1-p_1) + p_2 (1-p_2)} \right]^2}{(p_1 - p_2)^2}$, where $pˉ= \frac{p_1 + p_2}{2}$. In this equation, n=required sample size per group, p1=proportion in thrombolysed group (2.7% = 0.027), p2=proportion in late non-thrombolysed group (22.9% = 0.229), α=type I error (0.05 for 95% confidence), β=type II error (1 − power, e.g., 0.05 for 95% power), Z1−α/2≈1.96(standard normal deviate for 95% confidence), Z1−β≈1.645(standard normal deviate for 95% power). The calculation yielded approximately 68 patients per group (total = 136). The expected number of events was approximately 2 in the thrombolysis group and 16 in the late non-thrombolysed group, giving a pooled prevalence of ≈13%. This sample size also accounted for potential missing or incomplete data and ensured a low probability of Type II error.

Sampling technique and grouping

A non-probability sequential sampling technique was used to include all eligible patients presenting consecutively during the study period. Patients were divided into two groups:

The thrombolysis group, comprising patients who received streptokinase 1.5 million IU intravenously over 60 minutes during their current hospital admission. They were treated according to institutional and international STEMI management protocols.

Late presentation without thrombolysis group, comprising patients who did not receive thrombolytic therapy and presented >12 hours after symptom onset.

Possible selection bias inherent to this approach has been acknowledged and discussed as a study limitation.

Inclusion and exclusion criteria

Inclusion Criteria

Adult patients aged 18-75 years presenting with acute STEMI were eligible for inclusion in the study. STEMI was defined according to standard diagnostic criteria, including the presence of a new left bundle branch block (LBBB) or ST-segment elevation ≥1 mm in two or more contiguous ECG leads, in combination with elevated cardiac troponin I or T levels. This ensured that all enrolled patients had objective evidence of acute myocardial injury and ischemia. Eligible participants were consecutively enrolled using a non-probability sequential sampling technique to capture all patients meeting these criteria during the study period, thereby providing a representative sample of STEMI patients presenting to the hospital.

Exclusion Criteria and Data Handling

Patients were excluded if they underwent primary PCI instead of thrombolysis, had incomplete medical records, presented more than 24 hours after symptom onset, or had contraindications to thrombolytic therapy, such as a history of hemorrhagic stroke, active bleeding, recent major surgery, or known bleeding disorders. Bleeding complications were prospectively classified as major (intracranial or gastrointestinal bleeding requiring transfusion or a hemoglobin drop ≥5 g/dL) or minor (epistaxis, ecchymosis, or self-limiting bleeding). Baseline characteristics, including demographics, cardiovascular risk factors, and time to hospital presentation, were compared between groups. Potential confounders were adjusted in multivariate regression analyses to account for differences in age, gender, comorbidities (such as diabetes, hypertension, and smoking), and presentation timing, ensuring accurate assessment of thrombolysis outcomes.

Data collection procedure

Data were collected using a structured proforma by trained medical officers blinded to patient group allocation. Collected variables included demographics, cardiovascular risk factors, time from symptom onset to hospital presentation, thrombolysis details, and in-hospital outcomes.

**Table 1:** Details of Structured Proforma. ECG, electrocardiogram; LBBB, left bundle branch block; IHD, ischemic heart disease; MI, myocardial infarction; WHO, World Health Organization; CVD, cardiovascular disease; ICU, intensive care unit

Section	Variables Collected	Details/response options
Demographics	Age, gender, residence	Recorded from patient/attendant
Clinical risk factors	Hypertension, diabetes, smoking, dyslipidemia, family history of IHD	Yes/no
Clinical presentation	Symptom onset time, chest pain type, associated symptoms (dyspnea, sweating, syncope)	Recorded as reported
Diagnostic findings	ECG findings (ST-elevation ≥1 mm in ≥2 contiguous leads, new LBBB), cardiac biomarkers	Confirmed by the cardiology team
Treatment details	Thrombolysis given (yes/no), symptom onset–needle time	Recorded in hours
In-hospital outcomes	Mortality, recurrent ischemia, post-MI risk (WHO CVD ≥20%), cardiogenic shock, arrhythmias, major bleeding	As per diagnostic criteria
Resource utilization	Hospital stay, ICU admission and duration, inotropic support, total hospital cost, 30-day readmission	Recorded from hospital records

Inter-observer reliability was ensured by independent re-verification of 10% of records, with discrepancies resolved by consensus. Missing or incomplete data (<5% of total variables) were excluded using listwise deletion; sensitivity analyses confirmed no major effect on outcomes.

In-hospital outcomes

Outcomes included mortality, recurrent ischemia, post-MI risk (using WHO CVD risk charts [15,16]), cardiogenic shock, arrhythmias (ventricular tachycardia/fibrillation, atrial fibrillation, complete heart block), and major bleeding events. All outcomes were monitored throughout hospitalization and confirmed clinically and electrocardiographically by the cardiology team.

Data analysis

Data were entered and analyzed using SPSS version 26 (IBM Corp., Armonk, NY). Continuous variables, such as age and hospital stay, were expressed as mean ± standard deviation and compared between groups using independent-samples t-tests, after verifying assumptions of normality with the Shapiro-Wilk test and homogeneity of variances with Levene’s test. Categorical variables, including gender, cardiovascular risk factors, and in-hospital outcomes, were analyzed using chi-square tests or Fisher’s exact tests where appropriate. To account for potential confounding, multivariate logistic regression was applied for key in-hospital outcomes, adjusting for age, gender, diabetes, hypertension, smoking status, and time to hospital presentation; confounders were selected based on prior literature and variables with univariate p < 0.10. Model adequacy was assessed using the Hosmer-Lemeshow goodness-of-fit test, and multicollinearity was evaluated by variance inflation factors (VIF < 5). Hospital costs were standardized in Pakistani Rupees (PKR) using institutional billing data and adjusted for duration of stay to allow meaningful comparisons. All statistical tests were two-tailed, and a p-value < 0.05 was considered statistically significant.

Ethical considerations

The study was conducted in accordance with the Declaration of Helsinki. Ethical approval was obtained from the Institutional Review Committee of DHQ Hospital KDA, Kohat (No. 383, dated 21.12.2023). Written informed consent was obtained from all participants or their attendants. All data were anonymized and stored on password-protected institutional servers accessible only to the research team. Research data will be retained securely for one year post-publication, after which it will be permanently deleted to maintain confidentiality and ethical compliance.

## Results

As shown in Table 2, a total of 136 STEMI patients were included, with 68 patients in the thrombolysis group and 68 in the late non-thrombolysis group. The mean age was 57.9 ± 9.8 years in the thrombolysis group and 58.5 ± 10.4 years in the late non-thrombolysis group. Male predominance was observed in both groups, with 51 (75.0%) in the thrombolysis group and 50 (73.5%) in the late non-thrombolysis group. Hypertension was present in 36 (52.9%) vs 38 (55.9%), diabetes in 24 (35.3%) vs 28 (41.2%), and smoking in 30 (44.1%) vs 27 (39.7%) of patients, respectively. Dyslipidemia occurred in 22 (32.4%) vs 25 (36.8%), and family history of ischemic heart disease in 15 (22.1%) vs 17 (25.0%). There were no discernible variations between the groups in any of the clinical or baseline demographic factors.

**Table 2:** Baseline demographic and clinical characteristics Values are presented as mean ± standard deviation (SD) or number (%). Statistical comparisons used the t-test and chi-square (χ²) test. None were statistically significant (p > 0.05). IHD: ischemic heart disease

Variable	Thrombolysis (n=68)	Late non-thrombolysis (n=68)	Test	p-value
Age (years), mean ± SD	57.9 ± 9.8	58.5 ± 10.4	t = 0.36	0.72
Male, n (%)	51 (75.0)	50 (73.5)	χ² = 0.05	0.82
Hypertension, n (%)	36 (52.9)	38 (55.9)	χ² = 0.12	0.73
Diabetes, n (%)	24 (35.3)	28 (41.2)	χ² = 0.57	0.45
Smoking, n (%)	30 (44.1)	27 (39.7)	χ² = 0.33	0.57
Dyslipidemia, n (%)	22 (32.4)	25 (36.8)	χ² = 0.33	0.56
Family history of IHD, n (%)	15 (22.1)	17 (25.0)	χ² = 0.17	0.68

**Figure 1:**
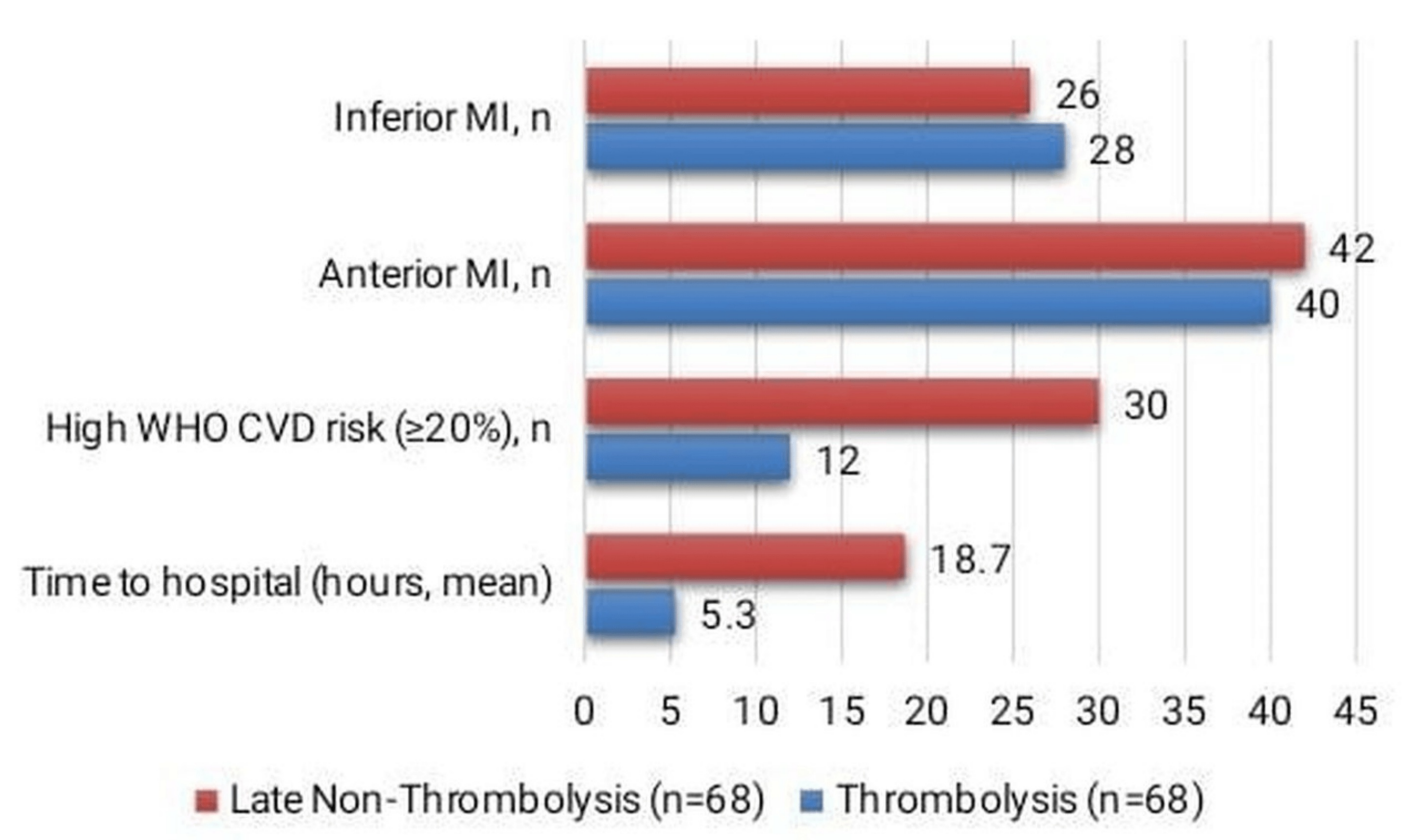
Time to hospital and clinical status at presentation Infarct location (anterior MI, χ² = 0.11, p = 0.73; inferior MI, χ² = 0.11, p = 0.73) was not significant. n = number of patients; % = percentage; SD = standard deviation; t = t-test; χ² = chi-square test. Values are presented as mean ± SD or number (%). Abbreviations: MI = myocardial infarction; WHO CVD = World Health Organization cardiovascular disease risk. High WHO CVD risk defined as ≥20% 10-year risk of a major cardiovascular event.

Patients in the thrombolysis group presented to the hospital significantly earlier, with a mean time of 5.3 ± 2.0 hours, compared to 18.7 ± 4.1 hours in the late non-thrombolysis group (t = 22.1, p < 0.001). As shown in Figure 1, a high WHO CVD risk (≥20%), indicating a worse clinical profile at admission, was observed in 12 patients (17.6%) in the thrombolysis group versus 30 patients (44.1%) among late presenters (χ² = 13.2, p < 0.001). Infarct location showed no significant differences between the groups, with anterior MI seen in 40 patients (58.8%) vs. 42 patients (61.8%) (χ² = 0.11, p = 0.73) and inferior MI in 28 patients (41.2%) vs. 26 patients (38.2%) (χ² = 0.11, p = 0.73). These findings indicate that delayed presentation is associated with higher WHO CVD risk at admission.

**Figure 2:**
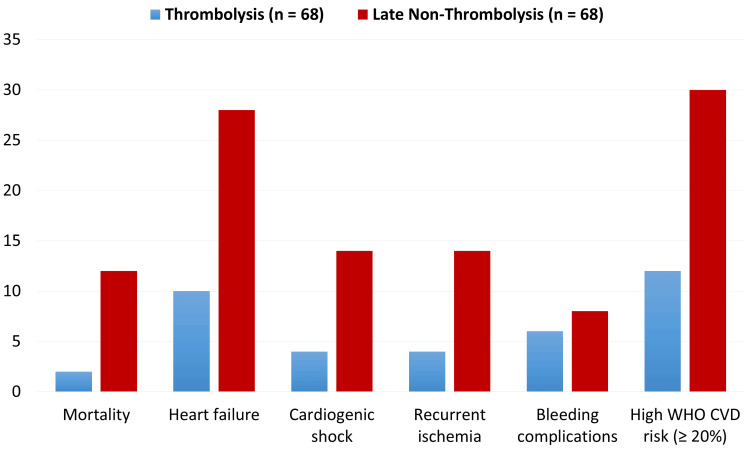
Comparison of major in-hospital outcomes between thrombolysis and late non-thrombolysis groups (n = 68 each).

Figure *2* compares the major in-hospital outcomes, including mortality, post-MI heart failure, recurrent ischemia, cardiogenic shock, arrhythmias, and bleeding complications, between patients who received thrombolysis (n = 68) and those who presented late and did not receive thrombolysis (n = 68). Adverse events were consistently lower in the thrombolysis group. Mortality (2.9% vs. 13.2%) and post-MI heart failure (11.8% vs. 27.9%) were markedly reduced among thrombolysed patients. The frequency of recurrent ischemia (5.9% vs. 20.6%) and cardiogenic shock (4.4% vs. 11.8%) was also lower. High WHO cardiovascular risk (≥20%) at admission was less common in the thrombolysis group (17.6% vs. 44.1%). Bleeding complications were rare and comparable between groups (minor bleeding: 8.8% vs. 11.8%). Overall, the figure demonstrates that thrombolysis was associated with significantly improved in-hospital outcomes, including lower mortality, fewer complications, and shorter hospital stay, without an increased risk of bleeding.

As shown in Table 3, bleeding events were generally low in both groups. Minor bleeding occurred in 6 (8.8%) patients in the thrombolysis group versus 8 (11.8%) in late non-thrombolysis patients (p = 0.57). No major bleeding events were reported. Bleeding complications were infrequent and comparable between groups (6 (8.8%) vs 8 (11.8%), p = 0.57. Gastrointestinal bleeding, hematuria, hematoma at the injection site, and thrombocytopenia were rare, occurring in ≤4.4% of patients in both groups. No hematological or hemorrhagic problems showed statistically significant differences between the groups. These findings suggest that thrombolysis is generally safe, even when compared to patients who present late without thrombolysis.

**Table 3:** In-hospital outcomes, complications, and resource utilization Values are presented as mean ± standard deviation (SD) or number (%). Statistical comparisons were performed using the t-test, χ² test, or Fisher’s exact test as appropriate. A p-value < 0.05 was considered statistically significant. Cost is reported in Pakistani rupees (PKR). Abbreviations: MI = myocardial infarction; WHO CVD = World Health Organization cardiovascular disease risk; ICU = intensive care unit; RD = risk difference

Variable	Thrombolysis (n=68)	Late non-thrombolysis (n=68)	Test	p-value	Absolute RD	95% CI / Mean ± SD
Primary outcomes						
Mortality, n (%)	2 (2.9)	9 (13.2)	χ² = 4.90	0.03	-10.3%	-18.0 to -2.5%
Post-MI heart failure, n (%)	8 (11.8)	19 (27.9)	χ² = 5.18	0.02	-16.1%	-28.1 to -4.1%
Recurrent ischemia, n (%)	4 (5.9)	14 (20.6)	χ² = 6.12	0.01	-14.7%	-26.4 to -3.0%
Cardiogenic shock, n (%)	3 (4.4)	8 (11.8)	χ² = 2.20	0.14	-7.4%	-17.0 to 2.2%
Major arrhythmia, n (%)	2 (2.9)	6 (8.8)	χ² = 2.11	0.15	-5.9%	-14.3 to 2.5%
Secondary outcomes						
Minor bleeding, n (%)	6 (8.8)	8 (11.8)	χ² = 0.32	0.57	-3.0%	-10.6 to 4.6%
High WHO CVD risk (≥20%), n (%)	12 (17.6)	30 (44.1)	χ² = 13.2	<0.001	-26.5%	-39.3 to -13.7%
Length of stay (days)	4.6 ± 1.2	6.5 ± 1.9	t = 6.4	<0.001	-1.9	95% CI 1.3–2.5
ICU admission, n (%)	5 (7.4)	13 (19.1)	Fisher	0.08	-11.7%	-23.6 to 0.2%
ICU stay (days)	1.4 ± 0.5	2.2 ± 0.8	t = 4.2	0.03	-0.8	95% CI 0.4–1.2
Inotropic support, n (%)	3 (4.4)	10 (14.7)	Fisher	0.06	-10.3%	-21.2 to 0.6%
Hospital cost (PKR)	45,200 ± 8,500	60,100 ± 13,200	t = 7.2	<0.001	-14,900	95% CI 11,000–18,800
30-day readmission, n (%)	1 (1.5)	5 (7.4)	Fisher	0.18	-5.9%	-13.1 to 1.3%

Patients receiving thrombolysis had shorter hospital stays (4.6 ± 1.2 days) compared to late non-thrombolysis patients (6.5 ± 1.9 days, p < 0.001). ICU admission was required in 5 (7.4%) vs 13 (19.1%) patients, while ICU stay duration was 1.4 ± 0.5 days versus 2.2 ± 0.8 days, both favoring thrombolysis. Inotropic support was needed in 3 (4.4%) versus 10 (14.7%) patients. Total hospital cost was significantly lower in the thrombolysis group (45,200 ± 8,500 PKR vs 60,100 ± 13,200 PKR, p < 0.001). Readmission within 30 days occurred in 1 (1.5%) versus 5 (7.4%) patients, showing a trend but not reaching significance. These results suggest that thrombolysis is linked to better clinical results and less use of medical resources.

Multivariate logistic regression confirmed that thrombolysis independently reduced composite adverse outcomes after adjusting for age, gender, comorbidities, and time to hospital (adjusted OR = 0.42; 95% CI 0.25-0.70; p = 0.001). As shown in Table 4, mortality was significantly lower in the thrombolysis group (OR 0.18, 95% CI 0.04-0.78, p = 0.02). Similarly, the odds of having high WHO CVD risk (≥20%) / post-MI heart failure were markedly reduced with thrombolysis (OR 0.28, 95% CI 0.11-0.69, p = 0.006). Recurrent ischemia was also significantly less frequent (OR 0.25, 95% CI 0.08-0.77, p = 0.02). Although the risk of cardiogenic shock (OR 0.30, 95% CI 0.07-1.27, p = 0.10) and major arrhythmias (OR 0.32, 95% CI 0.09-1.16, p = 0.08) was lower in the thrombolysis group, these associations did not reach statistical significance.

**Table 4:** Multivariate logistic regression for key outcomes Multivariate logistic regression adjusted for age, sex, hypertension, diabetes, smoking, and dyslipidemia. A p-value < 0.05 was considered statistically significant. Abbreviations: OR = odds ratio; CI = confidence interval; WHO CVD = World Health Organization cardiovascular disease risk; MI = myocardial infarction; HF = heart failure; EPV = events per variable

Outcome	Variable	Adjusted OR (thrombolysis vs late non-thrombolysis)	95% CI	p-value	EPV
Mortality	Thrombolysis vs. late non-thrombolysis	0.18	0.04–0.78	0.02	9
High WHO CVD risk (≥20%) / Post-MI HF	Thrombolysis vs. late non-thrombolysis	0.28	0.11–0.69	0.006	42
Recurrent ischemia	Thrombolysis vs. late non-thrombolysis	0.25	0.08–0.77	0.02	18
Cardiogenic shock	Thrombolysis vs. late non-thrombolysis	0.30	0.07–1.27	0.10	11
Major arrhythmia	Thrombolysis vs. late non-thrombolysis	0.32	0.09–1.16	0.08	8

## Discussion

This study demonstrates that timely thrombolysis in STEMI patients is associated with markedly better in-hospital outcomes compared to those who presented late and did not receive thrombolysis. Conducted at a single tertiary care center, the findings - though not universally generalizable - offer valuable insight into clinical management in settings where access to primary PCI remains limited. The sample size was adequate to evaluate primary endpoints; however, it may not have been sufficiently large to detect differences in relatively infrequent adverse events such as major bleeding or severe arrhythmias. Despite this limitation, the observed trends provide compelling evidence supporting the early use of thrombolytic therapy in acute myocardial infarction management.

Patients who received thrombolysis exhibited significantly lower mortality, fewer recurrent ischemic events, and a smaller proportion of individuals classified as high WHO cardiovascular risk compared with the late non-thrombolysis group. Furthermore, thrombolysed patients experienced shorter hospital stays, reduced ICU duration, and lower overall hospitalization costs. These findings underscore not only the clinical efficacy but also the resource utilization advantages of timely reperfusion. Importantly, bleeding and hematological complications were infrequent and did not differ significantly between groups, reinforcing the safety profile of thrombolysis when administered within the recommended therapeutic window.

The multivariate regression analysis further strengthened these observations by confirming that thrombolysis independently reduced adverse outcomes even after adjusting for age, gender, comorbid conditions, and time to hospital presentation. This suggests that the beneficial effects of timely thrombolysis are robust and persist beyond baseline risk differences. Collectively, these results indicate that early thrombolytic therapy provides a substantial protective effect against in-hospital morbidity and mortality, highlighting its value as a lifesaving intervention in STEMI care pathways.

When compared with existing literature, the present findings are in close agreement with earlier studies demonstrating that early reperfusion significantly improves survival and reduces post-infarction complications [17]. Prompt thrombolysis minimizes ischemic time, thereby preventing irreversible myocardial damage, decreasing the risk of left ventricular dysfunction, infarct expansion, and cardiogenic shock [18]. The observed reduction in high WHO CVD risk and recurrent ischemic episodes among thrombolysed patients corroborates the well-documented time-sensitive nature of reperfusion therapy and its critical role in preserving cardiac function [19]. These findings collectively reinforce the established “time is muscle” paradigm that underpins current STEMI management guidelines.

Additionally, the observed reductions in hospital stay length, ICU utilization, and the need for inotropic or mechanical support among patients receiving thrombolysis reflect faster recovery trajectories and more efficient healthcare resource use. Such outcomes are consistent with real-world studies that have reported substantial cost savings and improved clinical efficiency in settings prioritizing early thrombolytic therapy [20]. The rarity of bleeding events in both groups also supports the favorable safety profile of thrombolysis, particularly when patient selection is appropriate and administration protocols are carefully followed [21]. Moreover, the reduced frequency of arrhythmic complications observed in this study adds to the growing evidence supporting the cardioprotective benefits of early reperfusion [22].

Finally, the independent association between thrombolysis and improved clinical outcomes, even after statistical adjustment for confounders, underscores the critical importance of timely intervention-especially in low- and middle-income countries where PCI access is constrained [23,24]. In view of these findings, optimizing pre-hospital care systems to reduce delays, enhancing public awareness of early symptom recognition, and standardizing thrombolytic therapy protocols should be prioritized to strengthen STEMI management. Future research should further explore long-term outcomes, post-discharge cardiac function, and strategies combining thrombolysis with adjunctive pharmacologic or interventional therapies to maximize survival and recovery benefits.

Limitations and future suggestions

This study was conducted at a single center, which may limit the generalizability of its findings to broader populations. The use of non-probability sequential sampling may also introduce selection bias. While the sample size was sufficient to evaluate the primary outcomes, it may not have been large enough to detect differences in infrequent adverse events such as major arrhythmias or severe bleeding. Additionally, only in-hospital outcomes were assessed, and long-term follow-up post-discharge was not performed, limiting insights into the sustained effects of thrombolytic therapy on morbidity, mortality, and quality of life.

Future studies should adopt multicenter designs with larger and more diverse cohorts to enhance external validity and capture long-term clinical outcomes. Research should also focus on strategies to reduce pre-hospital delays, optimize the timing and selection of thrombolytic therapy, and investigate the combined use of thrombolysis with adjunctive pharmacologic or mechanical interventions. Such efforts are particularly important in resource-limited settings, where access to primary PCI is limited, to further improve STEMI care and patient outcomes.

## Conclusions

Thrombolysis in STEMI patients was associated with significantly better in-hospital outcomes compared to patients who presented late and did not receive reperfusion therapy. Early thrombolytic intervention reduced mortality, lowered the proportion of patients with high WHO CVD risk (≥20%) at admission, and decreased recurrent ischemic events, while also shortening hospital stay, reducing ICU admissions and duration, and lowering overall healthcare costs. Bleeding complications were rare overall, and their comparable frequency between groups supports the safety of thrombolytic therapy when administered within the recommended timeframe. We acknowledge that these findings are limited to in-hospital outcomes and do not capture long-term morbidity or mortality. The single-center design and sequential patient selection may limit generalizability and introduce selection bias. Observed cost reductions reflect direct medical costs in our setting and may differ in other healthcare systems or with alternative thrombolytic agents. Due to the comparative cross-sectional design, causation cannot be firmly established. Nonetheless, these results underscore the critical importance of early recognition and rapid reperfusion, and support interventions such as public education on symptom recognition and improved pre-hospital STEMI care systems. Future research should focus on long-term cardiac function, quality-of-life outcomes, and rehospitalization rates to better evaluate the sustained impact of thrombolysis.
